# Comparison of Coronary Artery Bypass Grafting and Drug-Eluting Stent Implantation in Patients With Chronic Kidney Disease: A Propensity Score Matching Study

**DOI:** 10.3389/fcvm.2022.802181

**Published:** 2022-04-01

**Authors:** Yang Li, XueJian Hou, TaoShuai Liu, Shijun Xu, Zhuhui Huang, XiaoYu Xu, Ran Dong

**Affiliations:** ^1^Beijing Anzhen Hospital, Capital Medical University, Beijing, China; ^2^Department of Pharmacy, Beijing Anzhen Hospital, Capital Medical University, Beijing, China

**Keywords:** coronary artery bypass grafting, percutaneous coronary intervention, chronic kidney disease, coronary artery disease, propensity score matching

## Abstract

**Objectives:**

To compare the long-term outcomes of coronary artery bypass grafting (CABG) vs. percutaneous coronary intervention (PCI) with drug-eluting stents (DESs) for coronary artery disease (CAD) patients with chronic kidney disease (CKD).

**Methods:**

Coronary artery disease patients with decreased kidney function (estimated glomerular filtration rate <60 ml/min/1.73 m^2^) who underwent CABG (*n* = 533) or PCI with DES (*n* = 952) from 2013 to 2020 were enrolled at a single center. The baseline characteristics and clinical outcomes were compared between the CABG and PCI groups for each matched pair of patients with CKD. The primary endpoint was the occurrence of all-cause death. The secondary endpoints were major adverse cardiovascular events (MACCEs) such as death, myocardial infarction (MI), stroke, and repeat revascularization.

**Results:**

A total of 1,485 patients underwent revascularization, such as 533 CABG and 952 patients with PCI. The median follow-up duration was 55.6 months (interquartile range 34.3–74.7 months). Multivariable Cox regression models were used for risk adjustment, and after propensity score matching (PSM), 399 patients were well matched in each group. The in-hospital mortality rate in the CABG group was higher than that in the PCI group, but the difference was not statistically significant (5.0 vs. 2.5%, *p* = 0.063). At the 1-year follow-up, CABG was associated with a lower survival rate than PCI (94.2 vs. 98.0%, hazard ratio [*HR*] of 3.72, 95% *CI* = 1.63–8.49, *p* < 0.01). At the end of the 5-year follow-up, the freedom from MI and the freedom from repeated revascularization were both better in the CABG group compared to the PCI group (89.1 vs. 81.7%, HR of 0.59, 95% *CI* = 0.38–0.92, *p* = 0.019; 86.9 vs. 73.8%, *HR* of 0.54, 95% *CI* = 0.36–0.81, *p* = 0.003, respectively). Furthermore, the freedom from MACCEs was also better in the patients of CABG compared with the patients of PCI (58.5 vs. 51.3%, *HR* of 0.71, 95% *CI* = 0.55–0.91, *p* = 0.030). CABG had a higher cumulative survival rate (68.4 vs. 66.0%) but without a statistically significant difference (*HR* of 0.92, 95% *CI* = 0.67–1.27, *p* = 0.602) compared with that of PCI.

**Conclusions:**

Compared to the use of PCI with a drug-eluting stent among patients with CKD, the use of CABG was associated with a lower MI rate, repeat revascularization rate, and lower number of MACCEs during the long-term follow-up. At a follow-up of 1 year, the number of MACCEs and other adverse events were comparable between the two cohorts, but CABG showed a lower survival rate than PCI.

## Introduction

The chronic kidney disease (CKD) population is increasing, and the CKD is associated with a higher threat to public health. In China, the incidence of CKD is ~10.8%, and the estimated number of patients with CKD is 119.5 million (1). The patients with CKD accounted for 4.8% of all hospitalized patients in China (2), and CKD may affect more than 10% of individuals worldwide (3). In the United States (4, 5), ~26 million Americans have CKD (6, 7), Nearly two-thirds of patients with CKD have coronary artery disease (CAD) (8). Cardiovascular disease is the major cause of death of patients with CDK (9, 10), and CKD also increases the risk of cardiovascular morbidity, mortality, and poor long-term outcomes (11).

The patients of CKD with CAD usually have multiple affected coronary arteries or left main stenosis (12). Both diseases simultaneously increase the risk of perioperative death and are associated with a worse prognosis after revascularization treatment (13). A decreased estimated glomerular filtration rate (eGFR) leads to worse outcomes from both coronary artery bypass grafting (CABG) and percutaneous coronary intervention (PCI) (11). However, prospective randomized controlled trials usually exclude patients of CAD with renal dysfunction. Most observational studies have excluded patients with CKD, and the optimal strategies for revascularization remain uncertain (14, 15).

This retrospective study analyzed the clinical results and long-term outcomes of patients with CKD who underwent CABG and PCI with DES in our center and aimed to compare the two revascularization methods and to improve patient prognosis.

## Methods

### Study Cohort

A total of 1,485 patients of CAD with CKD whose eGFR was <60 ml/min/1.73 m^2^ underwent CABG or PCI at a single hospital between 2013 and 2020. There were 533 patients who underwent CABG and 952 patients who received PCI with a drug-eluting stent. The inclusion criteria were adult patients (over age 18) who underwent CABG or PCI. The exclusion criteria to enter the study were: (1) age < 18, (2) incomplete data, (3) serious infection, (4) received kidney transplant, (5) history of surgical procedures (heart transplantation, ventricular assist device implantation, implantable cardioverter defibrillator), (6) preoperative ventilation, and (7) combined with valve replacement or other cardiac surgical procedure. The diagnoses of all of the patients were confirmed by the coronary angiography.

### Participation

The patient data were evaluated if the patient met the inclusion criteria after the preoperative examination. To ensure that the patients voluntarily participated in the study, during the follow-up, we contacted the family members or the patients, asked whether adverse events had occurred and carried out health education for the patients.

### Definition

The primary endpoint was the occurrence of all-cause death. The secondary endpoints included major adverse cardiovascular events (MACCEs) (defined as death, myocardial infarction [MI], stroke, or repeat revascularization). CKD was defined as eGFR <60 ml/min/1.73 m^2^, which was calculated using the Modification of Diet in Renal Disease (MDRD) equation that was modified from the original MDRD equation by adding a racial factor based on the Chinese population (16). The diagnosis of Acute myocardial infarction (AMI) required an increase or drop in troponin values and the presence of at least one of the following criteria: (1) symptoms of acute myocardial ischemia, (2) new ischemic electrocardiographic (ECG) findings, and (3) imaging evidence of loss of viable myocardium or abnormal motion of any of the walls due to an ischemic cause (17). Repeat revascularization included redo-CABG or PCI of the target vessel and other vessels. Stroke was diagnosed based on the imaging modalities by a neurologist.

### Revascularization Procedure

Coronary artery bypass grafting was performed using standard bypass techniques, such as on-pump and off-pump surgery, depending on the cardiac function and stenosis degree of the coronary artery. The left internal mammary artery was harvested for revascularization of the left anterior descending (LAD) artery whenever possible. The radial artery and saphenous vein were also harvested, if necessary, for complete revascularization. PCI was performed according to the current clinical guidelines (18). A drug-eluting stent (DES) was implanted in all patients, and a loading dose of 300 mg clopidogrel was given to all patients before PCI. After discharge, the patients in both groups received 100 mg aspirin daily for antiplatelet therapy, and clopidogrel or ticagrelor was used for the anticoagulation or antiaggregation therapy for 1 year in the CABG group and for at least 6 months in the PCI group unless severe bleeding occurred.

### Data Collection

The baseline characteristics included the demographics (age, body mass index, and sex), renal function (eGFR and serum creatinine), comorbidities (hypertension, diabetes, smoking, previous PCI, and stroke), cardiac status (left ventricular ejection fraction [LVEF], the number of coronary vessels with lesions, and left main disease, and New York Heart Association classification), and baseline laboratory findings (BUN, hemoglobin, triglyceride, and cholesterol). The surgical procedure included the surgical approach, number of grafts and stents, intra-aortic balloon pump (IABP) usage, and duration of surgery. The postoperative variables included the ICU and mechanical ventilation time, mortality, red blood cell transfusion, MI, stroke, reoperation for bleeding, new-onset dialysis or artificial fibrillation (AF), discharge medications, and cost.

The preoperative and perioperative data and clinical outcomes were retrospectively collected by the independent research personnel. All follow-up results were obtained by phone or Email from the patients themselves or their relatives at 1 year and 5 years after the procedure.

### Statistical Analysis

Continuous variables are described as the mean ± SD or the median and interquartile range (IQR) and were compared with Student's *t*-test or the Wilcoxon rank-sum test. Categorical variables were expressed as numbers and percentages, and the chi-square test was used according to the distribution. The propensity score matching (PSM) analysis was performed to account for the baseline differences. The matching conditions included age, sex, body mass index, smoking status, hypertension, diabetes, carotid artery stenosis, history of PCI, stroke, congestive heart failure, dialysis, eGFR, hemoglobin, LVEF, the number of diseased coronary vessels, and other variables. Logistic regression analysis was used to establish the CABG propensity score, which was then used for 1:1 nearest-neighbor matching with the PCI group. The effects of PCI compared to CABG for individual endpoints are expressed as *HRs* with 95% CIs. All tests were two-tailed, and *p* values < 0.05 were considered statistically significant. All statistical analyses were conducted by a statistician with SAS software version 9.4 (SAS Institute, Cary, NC, USA).

## Results

### Baseline Clinical and Procedural Characteristics Before PSM

The CABG group was older and had a higher percentage of men, a family history of CAD, carotid artery stenosis and a higher level of eGFR than the PCI group (*p* < 0.05). The PCI group had more patients with hypertension, diabetes, or previous atrial fibrillation and had higher hemoglobin, serum triglyceride, or cholesterol levels than the CABG group (*p* < 0.05). The number of diseased coronary vessels and the proportion of left main disease in the CABG group were higher than those in the PCI group (*p* < 0.05). There was no significant difference in the preoperative left ventricular ejective fraction between the two groups (*p* = 0.576). The rate of dialysis was 16.3 vs. 18.3% in the CABG and PCI groups, respectively, without a significant difference (*p* = 0.343) (Table 1).

**Table 1:** Comparison of preoperative baseline characteristics between CABG and PCI groups.

**Clinical variables**	**Before PS matched**		***p*** **-value**	**After PS matched**		***p*** **-value**
	**PCI**	**CABG**		**PCI**	**CABG**	
	**(*n* = 952)**	**(*n* = 533)**		**(*n* = 399)**	**(*n* = 399)**	
Age (years)	62.9 ± 12.26	64.9 ± 8.84	<0.001	64.3 ± 12.9	64.4 ± 9.1	0.937
Male (%)	700 (73.6)	446 (83.7)	<0.001	318 (79.7)	325 (81.1)	0.531
BMI (kg/m^2^)	26.08 ± 3.38	25.84 ± 3.25	0.185	26.1 ± 3.49	25.1 ± 3.28	0.887
Family history (%)	18 (1.9)	19 (3.6)	0.002	9 (2.3)	16 (4.0)	0.222
Hypertension (%)	811 (85.2)	398 (74.7)	<0.001	311 (77.9)	317 (79.4)	0.604
Diabetes (%)	441 (46.3)	187 (35.1)	<0.001	153 (38.3)	153 (38.3)	1.000
Smoking (%)	394 (41.4)	222 (41.7)	0.921	165 (41.1)	156 (39.1)	0.516
Heart failure (%)	45 (4.7)	13 (2.4)	0.030	17 (4.3)	9 (2.3)	0.162
COPD (%)	35 (3.7)	13 (2.4)	0.120	18 (4.5)	9 (2.3)	0.078
Carotid artery stenosis (%)	38 (4.0))	111 (20.8)	<0.001	34 (8.5)	37 (9.3)	0.709
ACS (%)	330 (34.7)	99 (18.6)	<0.001	92 (23.1)	85 (21.3)	0.551
Angina (%)	622 (65.3)	432 (81.1)	<0.001	307 (76.9)	314 (78.7)	0.730
Emergency surgery (%)	29 (3.0)	23 (4.3)	0.202	12 (3.0)	15 (3.8)	0.557
Previous MI >3 weeks (%)	347 (36.4)	164 (30.8)	0.027	137 (34.3)	119 (29.8)	0.172
Previous PCI (%)	219 (23.0)	61 (111.4)	<0.001	54 (13.5)	53 (13.3)	0.917
Previous atrial fibrillation (%)	58 (6.1)	17 (3.2)	0.014	26 (6.5)	10 (2.5)	0.006
Previous TIA or stroke (%)	143 (15.0)	68 (12.9)	0.231	59 (14.8)	43 (10.8)	0.090
Paralysis (%)	11 (1.2)	11 (2.1)	0.165	3 (0.8)	7 (1.8)	0.203
Hemoglobin (g/L)	119.9 ± 24.6	111.0 ± 21.33	<0.01	112.6 ± 21.2	110.8 ± 23.3	0.056
Serum albumin (mmol/L)	31.4 ± 7.4	41.7 ± 8.2	0.512	41.6 ± 7.3	41.7 ± 8.4	0.993
Triglyceride (mmol/L)	3.1 ± 2.5	1.8 ± 1.1	<0.01	2.2 ± 1.2	2.1 ± 1.8	0.742
Cholesterol (mmol/L)	4.5 ± 1.6	4.2 ± 1.0	<0.01	4.5 ± 1.1	4.7 ± 1.3	0.113
eGFR (ml/min/1.73 m^2^)	33.06 ± 16.89	34.81 ± 13.45	0.030	33.88 ± 16.22	34.03 ± 13.38	0.890
eGFR <30ml/min/1.73 m^2^	565 (59.3)	373 (70)	<0.01	258 (64.7)	267 (66.9)	0.502
Dialysis (%)	174 (18.3)	87 (16.3)	0.343	69 (17.3)	71 (17.8)	0.852
Left main disease (%)	93 (9.8)	90 (16.9)	<0.01	58 (14.5)	55 (13.8)	0.761
No.of narrowed coronary arteries	2.8 ± 0.79	3.1 ± 0.63	<0.01	3.0 ± 0.84	3.0 ± 0.63	0.962
≤ 2 (%)	358 (376)	83 (15.9)		113 (28.3)	71 (17.6)	
3 (%)	426 (44.7)	334 (62.7)		188 (47.1)	256 (64.2)	
≥4 (%)	168 (17.9)	116 (21.8)		98 (26.4)	72 (18.0)	
LVEF%	56.2 ± 11.4	56.6 ± 10.8	0.576	55.2 ± 11.38	56.7 ± 11.13	0.053
NYHA classification (%)			<0.01			0.450
1	72 (7.6)	16 (3.0)		20 (5.0)	12 (3.0)	
2	615 (64.6)	337 (63.2)		241 (60.40)	256 (64.2)	
3	199 (20.9)	149 (28.0)		111 (27.8)	106 (26.6)	
4	66 (6.9)	31 (5.8)		27 (6.8)	25 (6.3)	

*The data are shown as mean ± SD or n (%); SD, Standard deviation; CABG, Coronary artery bypass grafting; PCI, Percutaneous coronary intervention; eGFR, Estimated glomerular filtration rate; BMI, Body mass index; MI, Myocardial infarction; TIA, Transient ischemic attack; ACS, Acute coronary syndrome; LVEF, Left ventricular ejection fraction; NYHA, New York Heart Association*.

The average number of grafts or stents was 3.0 ± 0.88 in the CABG group and 2.8 ± 0.98 in the PCI group. In the CABG group, the mean operative time was 4.33 ± 1.05 h, and the left internal mammary artery was used in 93.1% of patients. Off-pump surgery was performed for 87.2% of patients. In the PCI group, coronary artery complications affected 1.23, and 0.6% of the serious patients were transferred to urgent or emergency CABG.

### Early Results Before PSM

The 30-day mortality rate in the CABG group was higher than that in the PCI group (7.1 vs. 2.1%, *p* < 0.01). In the CABG group, the incidence of perioperative complications, such as MI and new-onset dialysis, was much higher (8.1 vs. 3.0%, 10.3 vs. 4.8%, *p* < 0.01), and the IABP implantation rate was also higher (16.9 vs. 2.9%, *p* < 0.01) than that in the PCI group. For CABG, the mean ventilator support time was 41.5 ± 12.8 h, and the rate of reoperation for bleeding was 7.5% (Table 2).

**Table 2:** Comparison of postoperative data between CABG and PCI groups.

**Postoperative variables**	**Before PS matched**		***p*** **-value**	**After PS matched**		***p*** **-value**
	**PCI**	**CABG**		**PCI**	**CABG**	
	**(*n* = 952)**	**(*n* = 533)**		**(*n* = 399)**	**(*n* = 399)**	
ICU time (h)		53.9 ± 11.2			51.9 ± 9.8	
Mechanic ventilation time (h)		41.5 ± 12.8			39.8 ± 10.7	
IABP (%)	28 (2.9)	90 (16.9)	<0.01	10 (2.5)	64 (16.0)	<0.01
Red Blood cell transfusion (U)		3.0 ± 1.6			2.7 ± 1.4	
Reoperation for bleeding (%)		40 (7.5%)			26 (6.5%)	
Re-intubation (%)		13 (2.43%)			8 (2.0%)	
Wound complications (%)		21 (3.9%)			‘4 (3.5%)	
Myocardial infarction (%)	29 (3.0)	43 (8.1)	<0.01	9 (2.3)	25 (6.3)	0.005
Cerebral infarction (%)	51 (5.4)	19 (3.6)	0.118	20 (5.0)	11 (2.8)	0.099
New-onset dialysis (%)	46 (4.8)	55 (10.3)	<0.01	20 (5.0)	38 (9.5)	0.014
New-onset AF (%)	53 (5.6)	91 (17.1)	<0.01	22 (5.5)	65 (16.3)	<0.01
Cost ($)	8,790 ± 4,249	19,796 ± 10,369	<0.01	8,963 ± 4,369	19,591 ± 10,421	<0.01
In-hospital mortality (%)	20 (2.1%)	38 (7.1%)	<0.01	10 (2.5%)	20 (5.0%)	0.063
Medication at discharge (%)						
Aspirin	896 (96.3)	470 (95.1)	0.275	378 (97.2)	358 (94.7)	0.083
Clopidogrel/Ticagrelor	879 (94.5)	458 (92.7)	0.176	370 (95.1)	350 (92.6)	0.145
Beta-blockers	774 (83.2)	434 (87.9)	0.021	324 (83.3)	331 (87.6)	0.094
Statins	804 (86.5)	401 (81.2)	0.009	330 (84.8)	309 (81.7)	0.252
Nitrates	576 (61.9)	447 (90.5)	<0.01	245 (63.0)	338 (89.4)	<0.01

*The data are shown as mean ± SD or n (%), SD, Standard deviation; CABG, Coronary artery bypass grafting; PCI, Percutaneous coronary intervention; IABP, Intra-aortic balloon pump; AF, Atrial fibrillation*.

### Propensity-Matched Analysis

Propensity matching yielded 399 CABG and 399 PCI patients who were well matched in most of the baseline characteristics (Table 1). In the CABG group, the mean operative time was 4.27 ± 0.98 h, the mean number of grafts was 2.9 ± 0.85, and the proportion of off pump coronary artery bypass grafting (OPCABG) was 89.7%. For PCIs, the mean number of stents was 2.9 ± 0.97. The rates of in-hospital new-onset dialysis and perioperative MI were higher following CABG (9.5 vs. 5.0%, 6.3 vs. 2.3%, *p* < 0.05), but there were no significant differences in 30-day mortality between the two groups (5.0 vs. 2.5%, *p* = 0.063) (Table 2).

**Figure 1:**
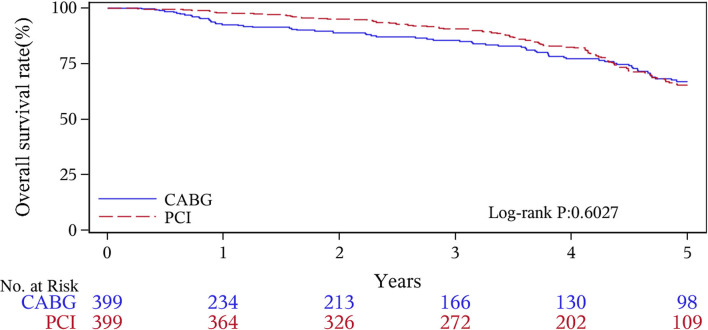
Death Kaplan–Meier (1–5 years).

**Figure 2:**
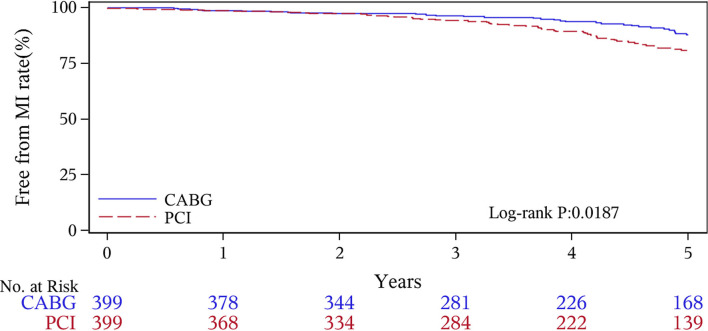
Myocardial infarction (MI) Kaplan–Meier (1–5 years).

**Figure 3:**
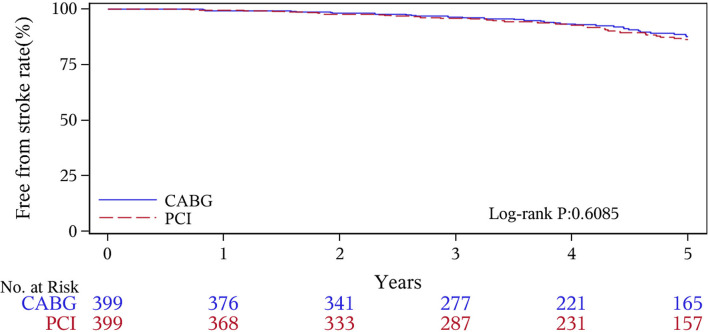
Stroke Kaplan–Meier (1–5 years).

**Figure 4:**
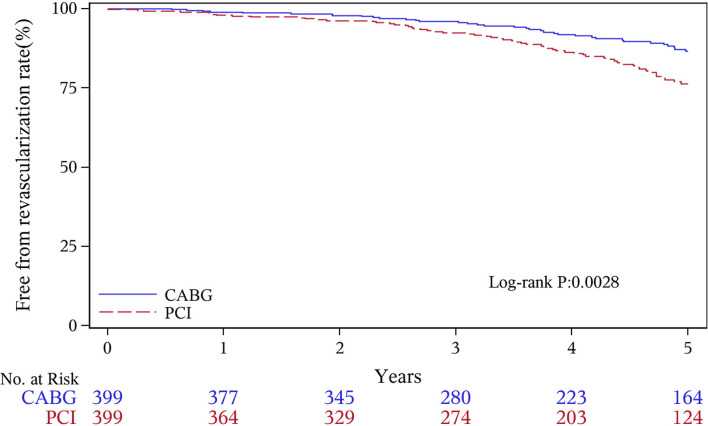
Revascularization Kaplan–Meier (1–5 years).

**Figure 5:**
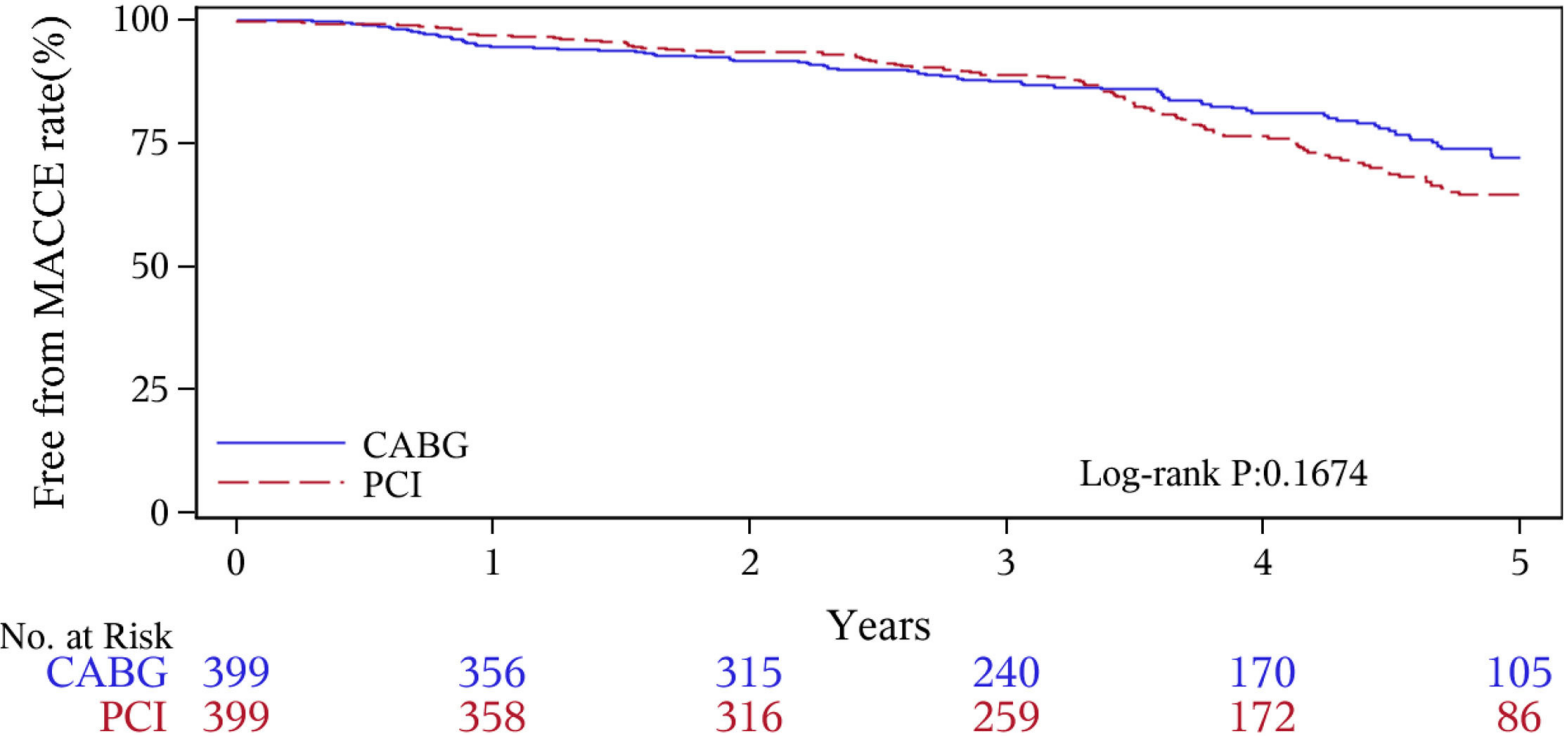
Major adverse cardiovascular event (MACCE) Kaplan–Meier (1–5 years).

The median follow-up duration was 55.6 months (IQR 34.3–74.7 months). During the 1 year of follow-up, in the CABG group, the survival rates were 94.2%; the freedom from MACCEs was 92.8%; the freedom from MI, stroke, or revascularization was 99.0, 98.0, and 98.7%, respectively. In the PCI group, the survival rates were 98.0%; the freedom from MACCEs was 95.9%; the freedom from MI, stroke, or revascularization was 98.4, 99.0, and 97.4%, respectively. At the end of 5-years follow-up, in the CABG group, the survival rates were 68.4%; the freedom from MACCEs was 58.5%; the freedom from MI, stroke, or revascularization was 89.1, 86.7, and 86.9%, respectively. In the PCI group, the survival rates were 66.0%; the freedom from MACCEs was 51.3%; the freedom from MI, stroke, or revascularization was 81.7, 91.3, and 73.8%, respectively (Figures 1–5).

During the 1-year follow-up, the CABG group had a higher hazard of mortality (*HR* of 3.72, 95% *CI* = 1.63–8.49), but the other adverse events between the two groups showed no significant differences, such as the freedom from recurrent MI (*HR* of 0.99, 95% *CI* = 0.29–3.43), the freedom from stroke (*HR* of 1.49, 95% *CI* = 0.25–8.92), the freedom from repeat revascularization (*HR* of 0.49, 95% *CI* = 0.15–1.64), and the freedom from MACCEs (*HR* of 1.76, 95% *CI* = 0.87–3.58). At the end of the 5-year follow-up, we found that, compared with PCI, CABG was associated with significantly lower risks for MI (*HR* of 0.59, 95% *CI* = 0.38–0.92), repeat revascularization (*HR* of 0.54, 95% *CI* = 0.36–0.81), and MACCEs (*HR* of 0.71, 95% *CI* = 0.55–0.91). The CABG group had a higher survival rate (*HR* of 0.92, 95% *CI* = 0.67–1.27) and a higher incidence of stroke (*HR* of 1.13, 95% *CI* = 0.71–1.81) than those in the PCI group, but the difference was not statistically significant (Table 3; Figure 1).

**Table 3:** Comparison of follow-up data between PCI and CABG groups after propensity score matching.

**End-points**	**CABG (***n*** = 399)**	**PCI (***n*** = 399)**	***p*** **-value**	**HR (95% CI)**
**Survival**
1-year	380 (94.2)	391 (98.0)	<0.01	3.72 (1.63,8.49)
5-year	333 (68.4)	310 (66.0)	0.602	0.92 (0.67,1.27)
**Freedom from myocardial infarction**
1-year	394 (99.0)	394 (98.4)	0.992	0.99 (0.29,3.43)
5-year	367 (89.1)	348 (81.7)	0.019	0.59 (0.38,0.92)
**Freedom from stroke**
1-year	396 (98.0)	397 (99.0)	0.659	1.49 (0.25,8.92)
5-year	367 (86.7)	363 (91.3)	0.608	1.13 (0.71,1.81)
**Freedom from repeat revascularization**
1-year	395 (98.7)	391 (97.4)	0.239	0.49 (0.15,1.64)
5-year	363 (86.9)	338 (73.8)	0.003	0.54 (0.36,0.81)
**Freedom from MACCEs**
1-year	379 (92.8)	387 (95.9)	0.112	1.76 (0.87,3.58)
5-year	324 (58.5)	307 (51.3)	0.030	0.71 (0.55,0.91)

*CABG, Coronary artery bypass grafting; PCI, Percutaneous coronary intervention; MACCEs, Main adverse cardiovascular and cerebrovascular events*.

### Subgroup Analysis

**Figure 6:**
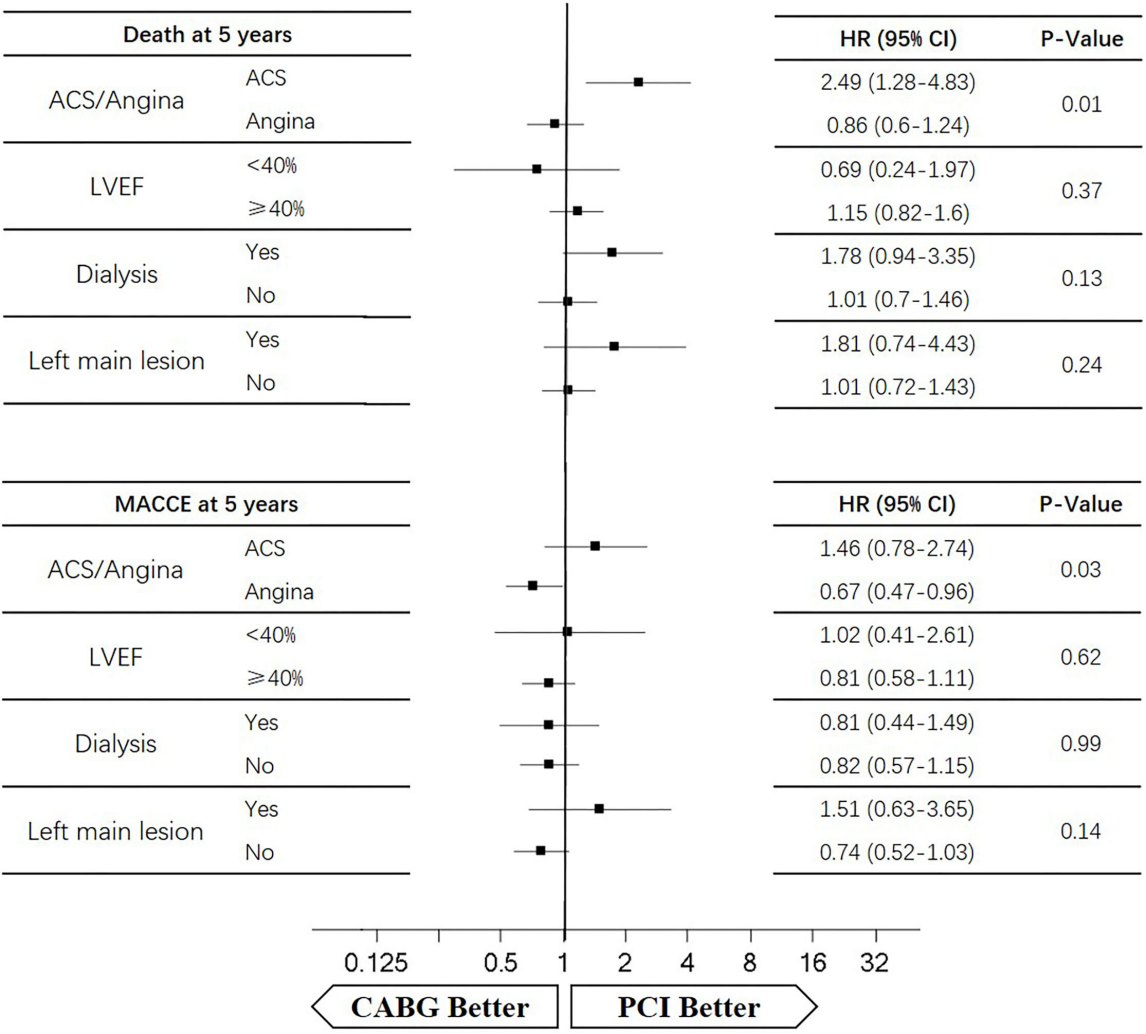
The 1-year death and MACCEs for patients according to subgroups.

**Figure 7:**
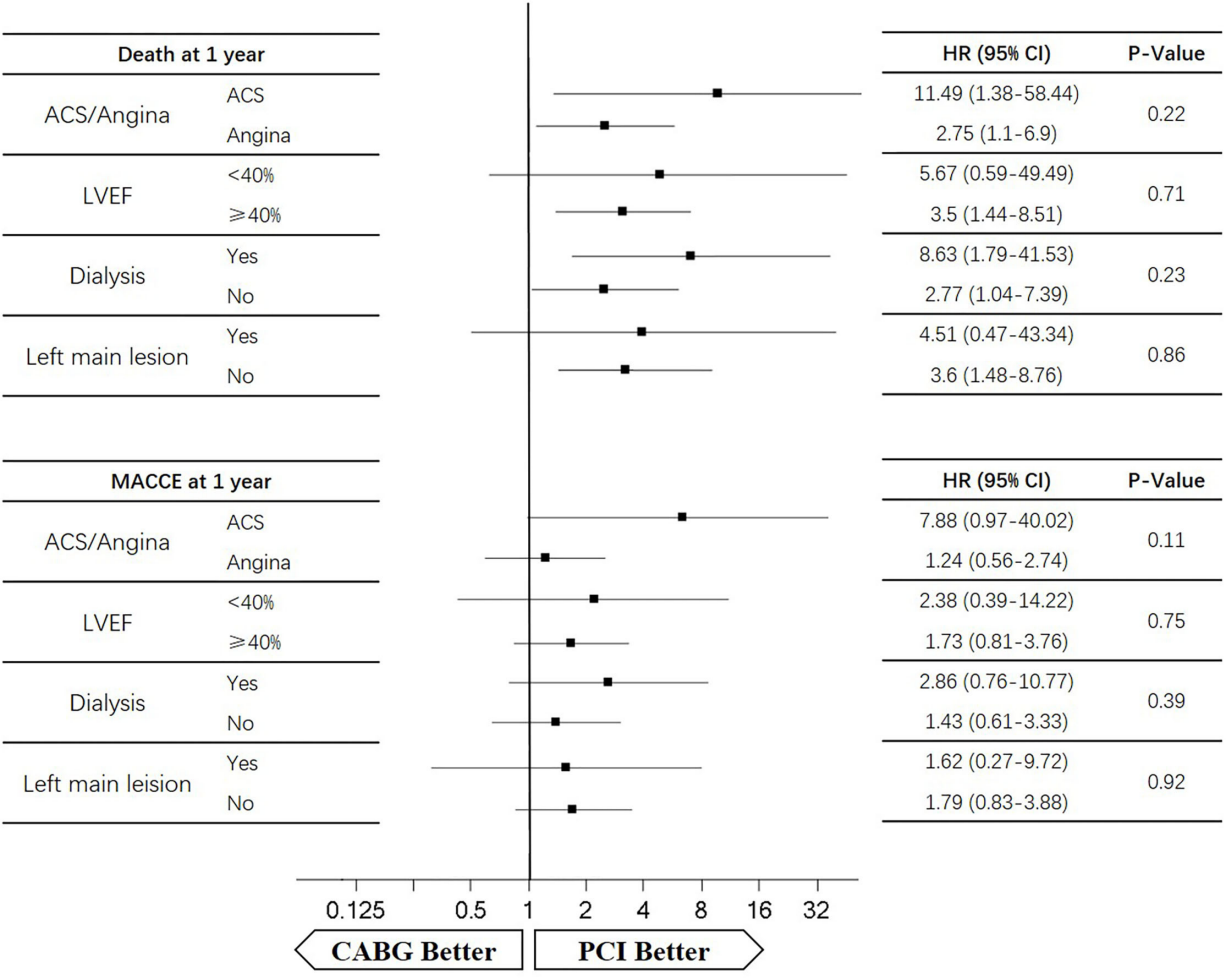
The 5-year death and MACCEs for patients according to subgroups.

Four subgroups were analyzed according to LVEF ≥ 40%, dialysis, left main lesion, and angina. Survival curves were created to estimate the effect of the two treatments in the whole cohort. In the angina patients, the cumulative 5-year survival rate was higher in the CABG group than in the PCI group (*HR* of 0.86, 95% *CI* = 0.6–1.24), whereas the incidence of MACCEs was significantly lower in the CABG group (*HR* of 0.67, 95% *CI* = 0.47–0.96). The cumulative 1-year survival rate was significantly lower in the CABG group than in the PCI group (*HR* of 2.75, 95% *CI* = 1.1–6.9), whereas the incidence of MACCEs was higher in the CABG group (*HR* of 1.24, 95% *CI* = 0.56–2.74) (Figures 6, 7).

In patients with LVEF <40%, the cumulative 5-year survival rate was higher in the CABG group than in the PCI group (*HR* of 0.69, 95% *CI* = 0.24–1.97), whereas the incidence of MACCEs was comparable between the two groups (*HR* of 1.02, 95% *CI* = 0.41–2.61). The cumulative 1-year survival rate was lower in the CABG group than in the PCI group (*HR* of 5.67, 95% *CI* = 0.59–49.49), whereas the incidence of MACCEs was higher in the CABG group (*HR* of 2.38, 95% *CI* = 0.39–14.22) (Figures 6, 7).

Among dialysis patients, the cumulative 5-year survival rate was lower in the CABG group than in the PCI group (*HR* of 1.78, 95% *CI* = 0.94–3.35), whereas the incidence of MACCEs was also lower in the CABG group (*HR* of 0.81, 95% *CI* = 0.44–1.49). The cumulative 1-year survival rate was lower in the CABG group than in the PCI group (*HR* of 8.63, 95% *CI* = 1.79–41.53), whereas the incidence of MACCEs was higher in the CABG group (*HR* of 2.86, 95% *CI* = 0.76–10.77) (Figures 6, 7).

In patients with left main lesion, the cumulative 5-year survival rate was lower in the CABG group than in the PCI group (*HR* of 1.81, 95% *CI* = 0.74–4.43), whereas the incidence of MACCEs was higher in the CABG group (*HR* of 1.51, 95% *CI* = 0.63–3.65). The cumulative 1-year survival rate was lower in the CABG group than in the PCI group (*HR* of 4.51, 95% *CI* = 0.47–43.34), whereas the incidence of MACCEs was higher in the CABG group (*HR* of 1.62, 95% *CI* = 0.27–9.72) (Figures 6, 7).

## Discussion

In this observational study of 1,485 patients of CAD with CKD, we found that at the 1-year follow-up, patients undergoing CABG had higher mortality but similar risks of MI, stroke, revascularization, and MACCEs compared to those who received PCI with DES implantation. During the long-term follow-up, the patients with CABG were associated with a reduced risk of MI, repeat revascularization, and MACCEs compared with the patients of PCI. The patients of CABG had a higher survival rate but there was no significant difference compared to the PCI group. The major causes of cardiovascular death included repeat MI and other induced complications, such as cardiac shock, malignant arrhythmia, heart failure, or fatal cerebral infarction, in both groups.

Coronary artery bypass grafting and PCI are the two currently used treatments for multivessel CAD, but which strategy is optimal for revascularization in patients with CKD is still unclear. Most randomized studies that have compared CABG with PCI excluded patients with CKD, so there is a lack of adequate evidence to confirm the curative effects of CABG (19, 20). Several clinical observational registries have found that CABG was superior to PCI in terms of its higher long-term survival rate and lower risks of revascularization and MI (21, 22). A meta-analysis of 18 studies reported that patients undergoing CABG had lower mortality and rate of MI, whereas the occurrence of stroke was much higher than that for PCI after more than 1 year (18.4 vs. 23.8%) (23). Two studies conducted by Weintraub WS and Hiroki Shiomi also revealed more benefits with CABG than with PCI for multivessel disease with a longer-term follow-up (24, 25). Baber (26) discovered in the FREEDOM trial that patients of CKD who received CABG had improved outcomes, such as lower rates of MI and repeat revascularization. Tara I. Chang analyzed 8,172 matched patients and found that CABG was associated with lower risks of death and acute coronary syndrome (ACS) than PCI (27). All of the results of these studies mentioned above were consistent with our 5-year follow-up results, suggesting that patients of CKD who received CABG showed a better prognostic trend than the PCI group and may preferentially benefit in terms of the long-term survival, and they avoided repeat revascularization or MI. The reasons leading to such results might be due to incomplete revascularization and higher rates of restenosis after PCI.

Although previously published trials favored CABG as a more beneficial revascularization strategy than PCI in the long-term follow-up outcomes, several studies have showed similar outcomes between the two methods regarding the early results. The Arterial Revascularization Therapies Study (ARTS) II study (28) showed that PCI was associated with no differences in mortality, stroke, or MI vs. CABG at the 1-year follow-up. A SYNTAX study also found comparable results for adverse events in patients with CKD who received CABG or PCI (29). In an analysis of 1,212 patients, CABG was associated with a reduced risk of MACCEs but an increased risk of cerebrovascular events, while there was no significant difference between the two groups regarding the all-cause mortality (30). Se Hun Kang et al. demonstrated that among patients with multivessel CAD and CKD, CABG resulted in similar rates of all-cause mortality, MI, or stroke but had a lower risk of repeat revascularization than PCI with DES (31). In the present study, we similarly found that the two groups exhibited no significant differences in prognosis at the end of the 1-year follow-up, except for a higher mortality in the CABG group.

The CABG strategy was associated with a better prognosis than PCI because the long-term outcomes might be attributed to the different preoperative baseline characteristics and to a more complete coronary artery vascularization. Some studies found that old age, emergency surgery, white race, heart failure, COPD, cerebrovascular diseases, female sex, CPB, and combined surgery were risk factors for the vascularization treatment (32). In our study, the patients in the PCI group were more likely to be women and had a higher proportion of smoking, COPD, and ACS; a higher history of heart failure, MI, or transient ischemic attack. Meanwhile, in the CABG group, all surgeries were simple bypass surgeries, off-pump surgery accounted for nearly 90%, their basic cardiac function was normal, and most surgeries were elective. Due to less dynamic perioperative fluid shifts, fewer red-cell transfusions, fewer bleeding complications, and a shorter mechanical ventilation time, off-pump CABG might have some benefits, such as a lower risk of developing ESRD, stroke, and a shorter hospital stay compared with the on-pump technique (33). It has been reported that OPCABG was more beneficial in patients with CKD (34), so the higher rate of off-pump surgery in our study might lead to more favorable results from CABG. Left ventricular systolic dysfunction had a poor prognosis especially for those whose LVEF <35–40% (35). The SCAAR (36) study revealed that in patients with ischaemic heart failure, long-term survival was greater after CABG than after PCI. Our sub-study for patients with LVEF <40% also found the similar results at 5-years follow-up.

In patients with a seriously decreased eGFR, the use of internal mammary artery grafts is an important protective factor to reduce the risk of operative death compared with venous grafts (37). In our study, nearly 95% of patients with CBAG had an internal mammary artery harvested for LAD, which might provide a better graft patency. The DESs were obviously improved with reduced coronary restenosis and repeated target vessel revascularization because of decreased neointimal proliferation (38). Despite the apparent improvements in the stent technology, the CABG strategy is still superior for patients with multivessel stenosis compared to PCI, and the advantages include a lower risk of mortality, MI and other events (39).

The patients with CKD are significantly associated with increased risks for mortality, MI, acute renal failure, and other adverse complications after both the PCI and CABG procedures (37). The patients with CKD have a higher rate of restenosis after PCI (40) and have a longer intubation retention time and hospital stay after CABG treatment. (37) Cooper et al. showed that the in-hospital mortality rate is inversely associated with a reduced eGFR, ranging from 1.3% in patients with normal kidney function to 9.3% in those with severe CKD (37). The patients with CKD usually have traditional risk factors for CAD, such as hypertension, hyperlipidemia, diabetes, and smoking. Simultaneously, due to oxidative stress, the increased rates of abnormal calcium, inflammation markers, phosphorus metabolism, vascular remodeling, and compliance are reduced, further accelerating the formation of atherosclerosis. In end-stage renal dysfunction (ESRD) patients, due to the increased media thickness and marked calcification, PCI is more challenging (1).

Our study has some other advantages over prior analyses. First, all of the surgeries were performed by the most skilled experts because our hospital is one of the largest centers for the treatment of CAD in China. Furthermore, our single-center database provided complete long-term follow-up results. Second, a large number of CKD patients need revascularization in China, but the relevant studies are rare. Our study can provide some experiences about the optimal revascularization strategies for Asians. Finally, DESs have been widely implemented worldwide, so the comparison between DESs and CABG is meaningful to reflect the optimal revascularization strategies.

There are some limitations of our study. First, this is a retrospective study, which may lead to potential selection bias, even though PSM cannot entirely obviate all bias. Second, this is a single-center non-randomized observation dataset, and the sample size of the patients of CKD might not be large enough, which may limit the ability to discern the differences and the accuracy of the analysis. Third, there is still a lack of clear revascularization guidelines for the patients of CKD. Finally, the long-term follow-up results require further analysis, and large registry trials are warranted.

In conclusion, the long-term clinical results of patients with CKD were better after CABG treatment because there was a lower risk of MI, repeat revascularization, and MACCEs, compared to PCI with DES. The survival rate was also higher for the patients of CABG than for the patients of PCI, but the difference was not significant. The 1-year survival rate was higher for patients of PCI, and the other adverse events were comparable between the two groups. Additional adequately powered randomized trials are needed to determine the optimal revascularization strategies for the patients of coronary heart disease (CHD) complicated by CKD.

## Data Availability Statement

The raw data supporting the conclusions of this article will be made available by the authors, without undue reservation.

## Ethics Statement

The studies involving human participants were reviewed and approved by Institutional Ethics Committee of Beijing Anzhen Hospital. The patients/participants provided their written informed consent to participate in this study. Written informed consent was obtained from the individual (s) for the publication of any potentially identifiable images or data included in this article.

## Author Contributions

YL and XH were responsible for the study concept and design. YL was responsible for drafting of the manuscript. All authors were responsible for the acquisition, analysis, or interpretation of data, critical revision of the manuscript for important intellectual content, read, and approved the final manuscript.

## Conflict of Interest

The authors declare that the research was conducted in the absence of any commercial or financial relationships that could be construed as a potential conflict of interest.

## Publisher's Note

All claims expressed in this article are solely those of the authors and do not necessarily represent those of their affiliated organizations, or those of the publisher, the editors and the reviewers. Any product that may be evaluated in this article, or claim that may be made by its manufacturer, is not guaranteed or endorsed by the publisher.
